# Evaluation of DISCOVAR *de novo* using a mosquito sample for cost-effective short-read genome assembly

**DOI:** 10.1186/s12864-016-2531-7

**Published:** 2016-03-05

**Authors:** R. Rebecca Love, Neil I. Weisenfeld, David B. Jaffe, Nora J. Besansky, Daniel E. Neafsey

**Affiliations:** Eck Institute for Global Health, University of Notre Dame, South Bend, IN 46556 USA; Department of Biological Sciences, University of Notre Dame, South Bend, IN 46556 USA; Genome Sequencing and Analysis Program, Broad Institute of MIT and Harvard, 415 Main St, Cambridge, MA 02142 USA

**Keywords:** *De novo* genome assembly, *Anopheles*, Heterozygosity, Insects

## Abstract

**Background:**

*De novo* reference assemblies that are affordable, practical to produce, and of sufficient quality for most downstream applications, remain an unattained goal for many taxa. Insects, which may yield too little DNA from individual specimens for long-read sequencing library construction and often have highly heterozygous genomes, can be particularly hard to assemble using inexpensive short-read sequencing data. The large number of insect species with medical or economic importance makes this a critical problem to address.

**Results:**

Using the assembler DISCOVAR *de novo*, we assembled the genome of the African malaria mosquito *Anopheles arabiensis* using 250 bp reads from a single library. The resulting assembly had a contig N50 of 22,433 bp, and recovered the gene set nearly as well as the ALLPATHS-LG AaraD1 *An. arabiensis* assembly produced with reads from three sequencing libraries and much greater resources. DISCOVAR *de novo* appeared to perform better than ALLPATHS-LG in regions of low complexity.

**Conclusions:**

DISCOVAR *de novo* performed well assembling the genome of an insect of medical importance, using simpler sequencing input than previous anopheline assemblies. We have shown that this program is a viable tool for cost-effective assembly of a modestly-sized insect genome.

**Electronic supplementary material:**

The online version of this article (doi:10.1186/s12864-016-2531-7) contains supplementary material, which is available to authorized users.

## Background

The rapid advances in genomics enabled by improvements in sequencing technology have demonstrated that a whole-genome reference assembly, whether in its own right or as a facilitator of population resequencing, is a valuable tool for answering biological questions in a diverse range of subfields. These include phylogenomics [1, 2], adaptation [3], ancient [4] and present-day [5] human demographics, molecular epidemiology [6], cancer biology [7], vector-borne disease control [8], and agriculture [9]. However, *de novo* genome assembly using short reads remains challenging for many species, especially those with highly heterozygous genomes.

Insects make up a large proportion of all known species [10], and may form an equally large proportion of undiscovered species [11]; they also include most pollinators [12], many species of medical importance, and many agricultural pests. The tendency of insects towards highly heterozygous genomes can, along with other factors, make their genomes difficult to assemble [13]. Current methods for creating quality genome assemblies, such as the use of long read technology [14, 15] or the generation of paired short sequence reads from fosmid-scale libraries [16], can be technically challenging and/or prohibitively expensive to accomplish with insects. For smaller insects, the large amount of DNA required to make a long-range library is often more than can be recovered from a single individual; DNA from multiple samples must be pooled, which introduces additional heterozygosity into the assembly process. In addition, extracting DNA of sufficient quality for the purpose of long-read sequencing or construction of long-range libraries requires starting with live samples, making it difficult to assemble genomes of species that must be collected from a remote locale. The creation of such libraries can be extremely labor intensive, with library construction costs exceeding sequencing costs by an order of magnitude or more. New long-read technology will help meet some of these challenges, but this solution may be costly as well. Better software may also have a role to play in producing decent genome assemblies from short read data.

*Anopheles arabiensis* is a major malaria vector of sub-Saharan Africa. Its recently-released reference genome, AaraD1, was assembled with 101 bp reads from three libraries, with insert sizes of 180 bp, 1.5 kb, and 38 kb, and exhibits contig and scaffold N50s of 74,117 bp and 5.6 Mb, respectively [17]. This level of assembly contiguity far exceeds the minimums recommended for gene annotation [13], but the production of two short-range and one costly long-range library will not scale well to large numbers of assembly projects; new assembly strategies are needed.

The recently-described genome assembler DISCOVAR *de novo* requires reads from only a single PCR-free library, and has tested well on relatively homozygous human and mouse genomes [18, 19]; we wished to test its potential for assembling an insect genome. If successful at assembling a comparatively polymorphic insect genome, DISCOVAR *de novo* could provide a simpler pathway to obtaining quality reference genomes.

We sequenced *An. arabiensis* and assembled its genome using DISCOVAR *de novo*. We then compared our assembly to the reference genome for this species in terms of contiguity, completeness, and gene recovery, with the goal of determining whether DISCOVAR *de novo* could produce a quality assembly from a single library of an insect species. We also examined the assembly for allelic variants predicted from the de Bruijn graph.

## Methods

### Library creation and sequencing

Using a CTAB protocol, DNA was extracted and pooled from 38 female sibs of *An. arabiensis* Dongola (MR4, NIAID/ATCC), the strain used for the creation of the ALLPATHS-LG reference assembly. From this extraction, a PCR-free library [20, 21] with 450 bp inserts was prepared (Table 1) from approximately 0.5 μg of DNA, in keeping with the input requirements of DISCOVAR *de novo*. The library was sequenced across two lanes of an Illumina HiSeq 2500 using paired-end 250 bp reads.

**Table 1:** Library specifications for inputs for *An. arabiensis* assemblies: the canonical assembly AaraD1 and the DISCOVAR *de novo* assembly

Assembly	Read length (base pairs)	Insert size (base pairs)
AaraD1	101	180;1500;38,000
D*dn*-Anara, raw and trimmed	250	450

### Assembly and processing

Two full lanes of sequencing produced coverage of approximately 664× (Additional file 1). Recommended coverage for DISCOVAR *de novo* (60×) is considerably less than this, so we made assemblies with data from half of one lane (121×), one lane (236×), and all of the data. Because it was closest to the recommended coverage, the assembly constructed using half of one lane (121×) was primarily used for all downstream analyses. Assemblies were produced with DISCOVAR *de novo* using default parameters.

We used nucmer [22] with default parameters to align the DISCOVAR *de novo* assembly (D*dn*-Anara) to the contigs of AaraD1, and removed all D*dn*-Anara contigs shorter than 2 kb; we also analyzed the impact of trimming the genome of contigs below 1, 3, 4, and 5 kb, to confirm that 2 kb was the optimum choice for this organism. We repeated the nucmer alignment after the trimming of D*dn*-Anara, and used GAEMR [23] to calculate basic assembly metrics before and after this trimming. We looked for low-complexity repeats in both assemblies, as well as sequence present in D*dn*-Anara in gaps between adjacent AaraD1 contigs aligned to D*dn*-Anara, using RepeatMasker [24] with the following parameters: -pa 4 -species anopheles -gccalc -lcambig -xsmall -poly.

### Scaffold analysis

One thousand one hundred of the 18,351 “contigs” present in the trimmed D*dn*-Anara assembly contained 100-bp-long sequences of *N*s, inserted to bridge small gaps in read coverage of indeterminate size spanned by read pairs. Though the D*dn*-Anara assembly incorporated no long-range scaffolding information, the resulting conglomerations of contigs were, technically, scaffolds. However, if these gaps were indeed limited to regions of approximately 100 bp, then the scaffolds in which they were contained might be more functionally equivalent to contigs than to traditional scaffolds.

To determine how well these 100-bp-long gaps in D*dn*-Anara approximated the actual distance between adjacent sequences, we split the putative scaffolds into their component contigs. Then we aligned these D*dn*-Anara component contigs to the AaraD1 contigs, the AaraD1 scaffolds, and the chromosomes of the *An. gambiae* PEST reference [25], using nucmer with default settings. We reduced this set of alignments to only those D*dn*-Anara scaffolds where each D*dn*-Anara contig had an unambiguous 1:1 hit with the reference used, and calculated the separation between alignments of adjacent D*dn*-Anara contigs to these references, using custom Python scripts.

### BUSCO analysis

To evaluate the quality and completeness of gene models contained in the D*dn*-Anara assembly, we used the set of universal single-copy benchmarking genes for arthropods described in [26] and calibrated them using *An. gambiae*, a member of the same species complex as *An. arabiensis*, as per the published protocol. We searched for the BUSCOs in D*dn*-Anara, the two DISCOVAR *de novo* assemblies constructed with higher coverage, the AaraD1 contigs, and the existing transcriptome assembly, using BLAST [27]. We also included the AaraD1 scaffolds to see how similar D*dn*-Anara was in functionality to an assembly created with a long-range library.

We processed the BLAST hits using Perl scripts as published in [26], and then further with custom scripts. We also used the blastn algorithm to search for all *An. gambiae* PEST (Agam4.2) protein-coding genes in these five assemblies, and processed the hits using custom Python scripts. The gene models of the PEST assembly, which is 10 years older than the AaraD1 assembly, were manually curated, and are expected to be more accurate and more complete than those in the *An. arabiensis* gene set (AaraD1.2).

### Relative performance of each assembler

To identify circumstances or genomic regions in which one assembler would outperform the other, we used the nucmer alignment between D*dn*-Anara contigs and AaraD1 contigs described above. Using awk, we identified AaraD1 contigs spanning multiple D*dn*-Anara contigs, and D*dn*-Anara contigs spanning multiple AaraD1 contigs, and filtered to remove very short or low-quality alignments. We extracted sequence spanned by a single D*dn*-Anara contig between multiple adjacent AaraD1 contigs, and looked for low-complexity sequence and repeats in this extracted sequence, using RepeatMasker with parameters as described above. We repeated this analysis with the two higher-coverage assemblies generated by DISCOVAR *de novo*.

To visualize regions where one assembler outperformed another, we aligned contigs from AaraD1 and from the DISCOVAR *de novo* assemblies to the PEST chromosomally-based reference assembly using nucmer with default parameters, and then generated a rough mapping scheme based on the longest contiguous or nearly-contiguous block of high-quality alignments.

### Genomic polymorphism

The pooled DNA template we used for library construction was derived from 38 full siblings, the parents of which were members of a partially inbred colony. We therefore anticipated that the number of haplotypes represented in the assembly would range from one to four, varying across the genome according to chance and selection against deleterious recessive alleles. To characterize the heterozygosity in our sequencing template and relate the performance of DISCOVAR *de novo* to this heterozygosity, we aligned raw sequencing reads to the D*dn*-Anara contigs using BWA [28] and called variants using GATK’s HaplotypeCaller walker [29, 30]. Variant positions were extracted and used to calculate variant density by contig. Contigs not present in the variant list were assumed to be homozygous.

One of the outputs of DISCOVAR *de novo* is a file representing genome polymorphism in a FASTA format enriched with “bubbles,” or forks in the de Bruijn graph. Each bubble contains the alleles predicted in the sequencing template at that locus (or, in the case of indels, one or more alleles and a gap). These alleles are later “flattened” into the final assembly.

A bubble denotes the presence of one or more variant positions, but the alleles within the bubble may extend past the variant position(s)—that is, this version of the assembly is locally phased. Using custom Python scripts, we sorted biallelic bubbles of 12.5 kb or less (for computational tractability) by type, into those consisting of exactly one single nucleotide polymorphism (SNP), exactly one indel, or a combination of SNPs and indels with interspersed invariant sequence. We then visualized contigs containing bubbles of exactly one SNP or exactly one indel using the PEST-based approach described above.

To contextualize the patterns observed, we also looked for putative separately-assembled haplotypes by aligning the D*dn*-Anara contigs to themselves, using BLAST with an e value of 1e-5 and other parameters set to default values. We visualized these putative separately-assembled haplotypes using the method described above.

## Results and discussion

### Basic assembly metrics

With DNA from a pool of 38 females, we used DISCOVAR *de novo* to construct a *de novo An. arabiensis* assembly (D*dn*-Anara). The resulting assembly had a contig N50 of 20,645 bp, and contained about 35 million bases not present in the contigs of the ALLPATHS-LG *An. arabiensis* reference assembly, AaraD1. (For consistency with the ALLPATHS-LG assembly, scaffolds shorter than 1 kb and contigs shorter than 200 bp were excluded from calculation of assembly statistics.) However, its total length was within a million bases of the length of the AaraD1 scaffolds (Tables 2 and 3).

**Table 2:** Basic assembly statistics and information for contigs in three *An. arabiensis* assemblies

Basic assembly statistics for contigs of three *An. arabiensis* assemblies						
Assembly	# Contigs	Contig N50 (bp)	Contig N90 (bp)	Mean Contig Length (bp)	Max Contig Length (bp)	Total Contig Length (bp)
AaraD1	10,179	74,117	10,908	20,772	819,682	211,443,117
D*dn*-Anara	29,408	20,645	3060	8364	271,474	245,971,991
D*dn*-Anara, trimmed at 2 kb	20,007	22,433	4364	11,631	271,474	232,710,450

**Table 3:** Basic assembly statistics and information for scaffolds in three *An. arabiensis* assemblies

Basic assembly statistics for scaffolds of three *An. arabiensis* assemblies						
Assembly	# Scaffolds	Scaffold N50 (bp)	Scaffold N90 (bp)	Mean Scaffold Length (bp)	Max Scaffold Length (bp)	Total Scaffold Length (bp)
AaraD1	1214	5,604,218	201,951	203,104	16,348,944	246,567,867
D*dn*-Anara	27,752	27,170	3062	8869	348,795	246,137,591
D*dn*-Anara, trimmed at 2 kb	18,351	30,033	4365	12,690	348,795	232,876,050

**Fig. 1:**
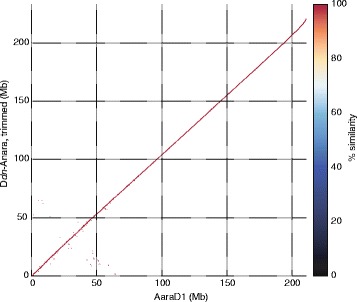
Alignment of trimmed D*dn*-Anara assembly to the AaraD1 assembly. Nucmer alignment of D*dn*-Anara contigs to AaraD1 contigs shows that nearly all sequence in the ALLPATHS-LG assembly is retained in D*dn*-Anara, even when contigs shorter than 2 kb are removed

Aligning the D*dn*-Anara “contigs” (see below) to the contigs of AaraD1 revealed that the D*dn*-Anara contigs that did not align were some of the shortest contigs in the assembly (Additional file 2). Short contigs (those less than 2 kb) were also disproportionately enriched for repeats/low-complexity sequence (Additional file 3), so we removed them for all downstream analyses. Aligning this trimmed D*dn*-Anara assembly to AaraD1 (Fig. 1) indicated that the trimmed assembly still retained nearly all the sequence of AaraD1. The trimming process also screened for contamination by removing essentially all contigs unique to D*dn*-Anara with significant hits in nt. We further investigated the impact of the trimming process to quantify the amount of genic sequence removed, and explore other possible trimming lengths, evaluating assemblies trimmed at five lengths total. We found that past the cutoff of 2 kb, the number of genes recovered in the assembly declined, as did the percentage of each gene recovered (Additional file 4) After trimming at the 2 kb cutoff, the contig N50 of the D*dn*-Anara assembly was 22,433.

The D*dn*-Anara assembly was not formally scaffolded with long-range information from additional libraries; instead, DISCOVAR *de novo* introduced 100-bp sequences of Ns to fill small gaps (see Additional files 5 and 6 for true size of filled gaps). Because the vast majority of these gaps were less than 500 bp, and the total gapped length was less than 200 kb (Additional file 7), we treated the D*dn*-Anara “scaffolds” as contigs for trimming and for all downstream analyses. (Calculated basic assembly statistics, however, maintain the scaffold-contig distinction.) The scaffold N50 of the (trimmed) D*dn*-Anara assembly was 30,033 bp. Basic assembly metrics for the D*dn*-Anara assemblies and AaraD1 are shown in Tables 2 and 3.

We also assessed the contiguity of the assemblies made with larger fractions of the total reads sequenced, before and after removing contigs shorter than 2 kb (Additional file 8); contig N50 for the assembly made from all sequenced reads was 32,261, nearly 10 kb longer than that of the assembly made from one half of one lane’s worth of reads and nearly half the contig N50 of the ALLPATHS-LG assembly. The scaffold N50 for this assembly was 51,707 bp; while we did not repeat the analysis of gaps (see above) for the assembly made with all the reads, if the pattern of the lowest-coverage assembly holds, these “scaffolds” contain only short gaps and function more like contigs. These basic assembly metrics suggest that significant increases in genome contiguity, if required, could potentially be obtained by increasing sequencing depth.

### Gene recovery

**Fig. 2:**
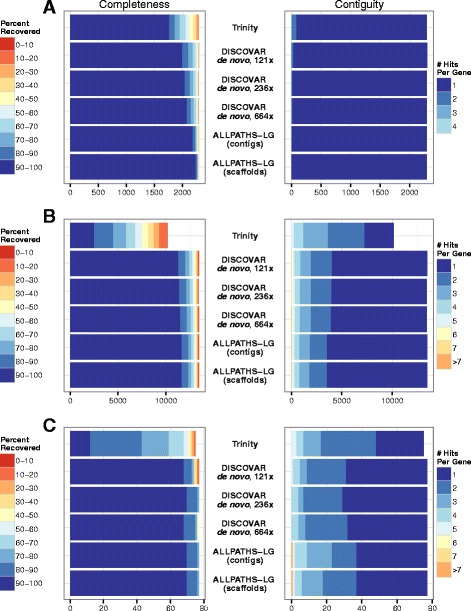
Analyzing the quality of 5 *An. arabiensis* genome or transcriptome assemblies in terms of gene content. We looked for three categories of genes in the Trinity transcriptome, D*dn*-Anara (labeled here as the 126× DISCOVAR *de novo* assembly), the higher-coverage DISCOVAR *de novo* assemblies, the ALLPATHS-LG AaraD1 contigs, and the ALLPATHS-LG AaraD1 scaffolds. **a** Benchmarking universal single-copy orthologs (BUSCOs) from the *An. gambiae* PEST genome. **b** All PEST genes, excluding 5′ and 3′ untranslated regions (UTRs). The result of using whole genes can be seen in the genes not recovered from the Trinity assembly. **c** Cuticle protein genes from low-complexity gene families identified in Cornman et al. 2009

*De novo* genome assembly is often a prelude to gene annotation or other gene-based analyses. To quantify the suitability of D*dn*-Anara for gene-based approaches, we searched the assemblies for members of a set of universal, single-copy arthropod genes (BUSCOs, [26]) in D*dn*-Anara, the contigs and scaffolds of AaraD1, and a transcriptome previously assembled with Trinity [17]. The BUSCOs were found with nearly equal completeness and contiguity in the contigs of AaraD1 and D*dn*-Anara, while BUSCOs found in the AaraD1 scaffolds were more complete and contiguous than in any set of contigs. Nearly all the BUSCOs were present in the Trinity assembly, but they were less complete, and more fragmented (Fig. 2a). Mean percent recovery from the AaraD1 contigs was 98.1 %, with each gene, on average, in 1.004 pieces; for the AaraD1 scaffolds, an average of 99.0 % of each gene was recovered, in 1.001 pieces. For D*dn*-Anara, the corresponding numbers were 95.9 % and 1.014 pieces. Increasing sequencing coverage resulted in modest improvements in the quality of gene models found in the DISCOVAR *de novo*-built assemblies: on average, 96.3 % of each gene was recovered from the whole-lane assembly, in 1.01 pieces. The assembly made with all reads recovered an average of 96.8 % of each gene, in 1.009 pieces.

When using nucleotide sequences of all protein-coding genes (AgamP4.2) from *An. gambiae*, a member of the same complex as *An. arabiensis* (Fig. 2b), differences between the AaraD1 contigs and scaffolds diminished. Mean percent recovery for AaraD1 contigs was 95.1 %, with each gene in an average of 1.50 pieces, while mean percent recovery for the scaffolds was 95.2 %, with the same contiguity. Mean percent recovery for D*dn*-Anara was 94.1 %, with each gene in an average of 1.56 pieces. Increasing the number of reads provided to DISCOVAR *de novo* again resulted in very modest improvements in completeness, but not contiguity; mean percent recovery for the one-lane assembly was 94.4 %, with each gene in, on average, 1.56 pieces, and for the assembly made with all reads, the corresponding numbers were 94.7 % and 1.556 pieces. For both gene sets, D*dn*-Anara closely approached not only the AaraD1 contigs but also the AaraD1 scaffolds in contiguity and completeness.

### Low-complexity sequence

**Fig. 3:**
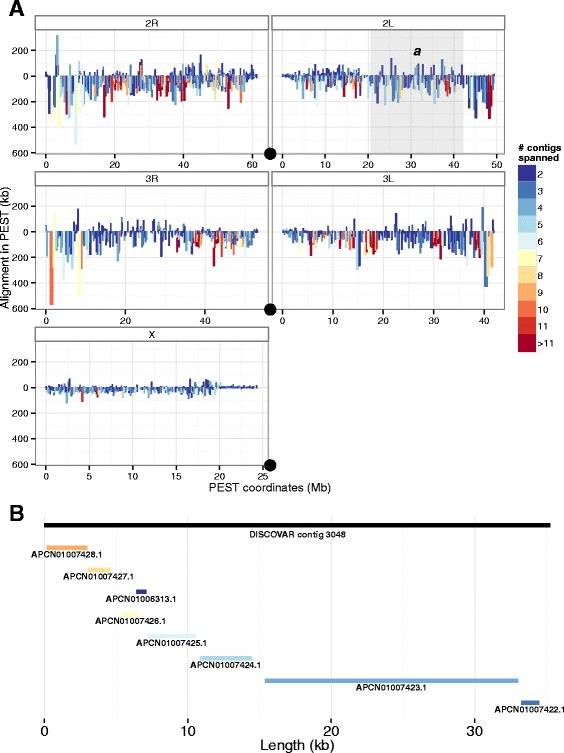
Assessing regions where one assembler outperforms the other. **a** *An. gambiae* chromosomal coordinates, and alignment length in that coordinate system, of AaraD1 contigs spanning multiple D*dn*-Anara contigs (*oriented downward*) and D*dn*-Anara contigs spanning multiple AaraD1 contigs (*oriented upward*). Centromeres are marked with *black circles*. Chromosomal inversion 2L*a*, which is fixed in *An. arabiensis* but polymorphic in *An. gambiae*, is indicated by a *grey box*. **b** Close-up of a D*dn*-Anara contig spanning the entirety of eight AaraD1 contigs, at approximately 24.1 Mb on the X chromosome

As expected from their respective contig N50s and general levels of contiguity, there are approximately twice as many instances of AaraD1 contigs spanning multiple D*dn*-Anara contigs (1905 contigs) than the reverse (1066 contigs) (Fig. 3a). However, D*dn*-Anara contigs spanning multiple adjacent AaraD1 contigs, though relatively short, seemed to concentrate in the centromeric regions. In these same regions, which tend to have high concentrations of low-complexity sequence, AaraD1 contigs spanning multiple D*dn*-Anara contigs occurred less frequently than in the rest of the genome. The sequence found in the D*dn*-Anara assembly between adjacent AaraD1 contigs was enriched for low-complexity sequence compared to the rest of the genome (Additional file 3), suggesting that DISCOVAR *de novo* might have an advantage over ALLPATHS-LG in assembling low-complexity sequence. One example of a D*dn*-Anara contig spanning seven adjacent AaraD1 contigs is shown in Fig. 3b.

We also saw this trend between the two assemblers when we focused on low-complexity cuticle protein genes identified in [31] (Fig. 2c). As in the whole gene set, AaraD1 contigs recovered more of each low-complexity gene than did D*dn*-Anara (a mean of 98.4 % for AaraD1 contigs and scaffolds, and 95.8 % for D*dn*-Anara); however, D*dn*-Anara recovered the low-complexity genes in fewer pieces than AaraD1 (an average of 1.96 pieces for AaraD1 contigs, 1.87 pieces for AaraD1 scaffolds, and 1.60 pieces for D*dn*-Anara). Together, these observations suggest that DISCOVAR *de novo* may have an advantage over ALLPATHS-LG in low-complexity regions.

We also examined performance in regions of low complexity in the assemblies made from one and two lanes of data. The assembly made by DISCOVAR *de novo* from one lane recovered low-complexity genes with better contiguity and completeness than any other assembly; an average of 98.4 % of each gene model was present in this assembly, in an average of 1.52 pieces. (Corresponding numbers from the assembly made with two lanes are 97.7 % and 1.57 pieces.) Increasing sequencing depth increased the number of DISCOVAR *de novo*-generated contigs spanning multiple AaraD1 contigs, and decreased the number of AaraD1 contigs spanning multiple DISCOVAR *de novo*-generated contigs (Additional file 9). The degree of improvement in centromeric regions was rather stochastic, but was most noticeable in the centromeric region of the X chromosome (Additional file 10).

### Genome-wide patterns of polymorphism

We characterized variation in the heterozygosity of our template by mapping sequencing reads back to the assembled DISCOVAR *de novo* contigs and calling variants. We found that while 39 % of the Ddn-Anara assembly is represented by contigs exhibiting no variants, the other 61 % of assembly exhibits a mean heterozygosity rate of 3.66 SNPs/kb, or 1 SNP every 273 bases. This rate of heterozygosity is approximately four times as high as that in humans [32]. This profile of partitioned heterozygosity, a product of the inbreeding history and pooled composition of the sequencing template, allows us to examine the performance of DISCOVAR *de novo* in both monomorphic and polymorphic compartments of the *An. arabiensis* genome.

**Fig. 4:**
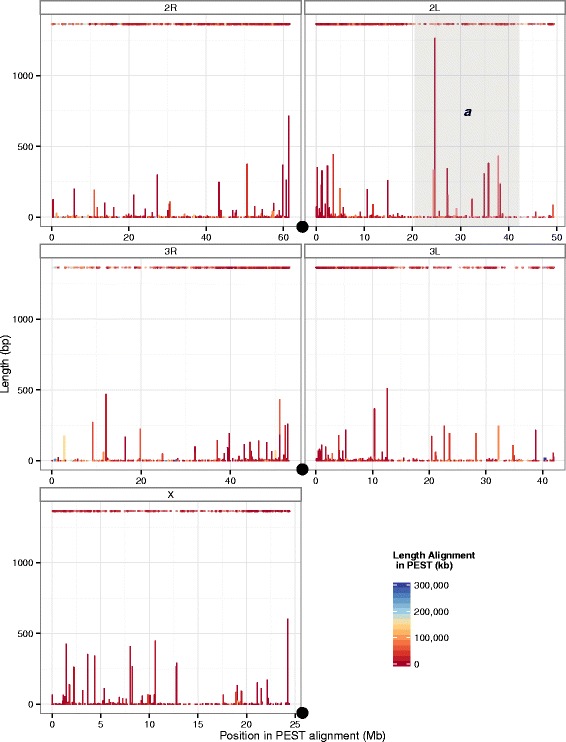
Genome-wide polymorphism. Locations, in PEST coordinates, of D*dn*-Anara contigs containing “bubbles” of exactly one SNP or exactly one indel. SNPs are designated by points at the *top* of the plot; indels are designated by *vertical lines*, anchored at the x-axis, corresponding to the length of the indel. Both types of variants are colored by the length in PEST coordinates of the contig on which they are found. Centromeres are marked with *black circles*

We also analyzed the variants predicted in the unflattened version of the assembly. The largest category of “bubbles” present within the assembly, before flattening, were 1 bp long, or traditional SNPs (Additional file 11). Contigs containing these bubbles, as well as contigs containing simple indels, concentrated near the centromeres of the autosomes, but were found throughout the genome (Fig. 4). To avoid overplotting, we identified and removed putative separately assembled haplotypes, contigs that aligned very well to another, similarly-sized contig over nearly all the length of both contigs. (While these contigs were shorter than the set of all contigs as a whole (Additional file 12), they showed levels of repetitive sequence similar to the rest of the genome (Additional file 3); this suggests that the similarity within each pair of contigs was not due to extended regions of low complexity, which further suggests that these contigs represent separately assembled haplotypes.) For many insect species, it may be necessary to pool samples to obtain sufficient DNA for library preparation (generally a minimum of 0.5 μg); in those cases, this approach of analyzing haplotype data may be generalizable to the identification and visualization of structural variation.

## Conclusions

Using reads from a single sequencing library, DISCOVAR *de novo* produced an *An. arabiensis* assembly with contig N50 of 22,433. This was substantially shorter than the 74,117 bp contig N50 of the ALLPATHS-LG *An. arabiensis* reference genome. However, if the D*dn*-Anara “scaffolds” are considered more like contigs than true scaffolds, given the small and sparse nature of the gaps they span, then a better measure of the contiguity of the D*dn*-Anara assembly is the scaffold N50 of 30,033. Results from assemblies made with higher coverage suggest that more contiguous assemblies could potentially be created, if necessary, simply by increasing the amount of sequence used as input to DISCOVAR *de novo*.

Despite the difference in contiguity between the D*dn*-Anara assembly and the ALLPATHS-LG reference assembly, the two assemblies performed similarly in terms of gene recovery, suggesting that D*dn*-Anara is sufficiently complete and contiguous to be used for virtually all downstream analyses based on the gene set. The assemblies made with higher coverage showed only modest improvements in gene recovery, possibly due to the already-high performance of D*dn*-Anara in this area.

While AaraD1 contigs tended to span multiple D*dn*-Anara contigs, as expected from their relative contig N50s, dramatic instances of the reverse were located in low-complexity regions of the genome near centromeres. D*dn*-Anara also assembled members of a low-sequence complexity gene family more contiguously than ALLPATHS-LG. Additionally, sequence spanned by D*dn*-Anara contigs but not AaraD1 contigs, was enriched for low-complexity sequence compared to the rest of the genome. Together, these findings suggest that DISCOVAR *de novo* may have an advantage over ALLPATHS in assembling regions of low-complexity sequence.

From one library of multiple individuals, DISCOVAR *de novo* produced an assembly functionally equivalent, for most gene-related purposes, to the reference assembly, AaraD1. In comparison, AaraD1 was assembled with ALLPATHS-LG from libraries of 180 bp, 1.5 kb, and 38 kb insert sizes; the 180 bp and 1.5 kb insert size libraries, from which the contigs were assembled, came from a single individual. AaraD1 was also reference-assisted with the high-quality, chromosomally-mapped genome of its close relative, *An. gambiae*. In light of the large differences in effort required to make each assembly, their similar performance suggests DISCOVAR *de novo* does well in balancing sequencing cost and assembly effort with resulting assembly quality.

The present study was limited to lab-bred individuals, to provide a fair comparison to the existing *An. arabiensis* assembly, which was made from the same strain. Generation of additional sequence will be required to directly assess the quality of assemblies made from wild-caught individuals. However, our results suggest that DISCOVAR *de novo* is a significant improvement over existing options to create a quality assembly from one sequencing library. DISCOVAR *de novo* has the potential to create, from relatively inexpensive sequencing libraries and read coverage, assemblies that are sufficiently complete and contiguous to serve a wide range of downstream comparative, population, and functional genomic analyses. Though DISCOVAR *de novo* has now been evaluated with a small, heterozygous genome (*An. arabiensis*), and for large, less heterozygous genomes (human and mouse) it remains to be tested against genomes that are both large and heterozygous, a combination of attributes common to many insects and other arthropods. Further work will be required to refine the scope of genomes appropriate for assembly with DISCOVAR *de novo*, but the present work suggests that genomes less than than 500 Mb may be successfully assembled from a single inexpensive library with this tool.

### Availability of data and materials

All sequencing reads generated for the DISCOVAR *de novo* assembly described in this manuscript have been deposited in the NCBI Sequence Read Archive under BioProject PRJNA304755.

## Additional files

Additional file 1: Sequencing output. Approximate coverage for three versions of D*dn*-Anara. (PDF 4 kb) Additional file 2: Contig size vs. alignment status. This plot shows that the D*dn*-Anara contigs that did not align to AaraD1 are some of the shortest in the assembly. (PDF 5 kb) Additional file 3: Sequence complexity. This table shows the proportion of bases masked in regions of D*dn*-Anara of particular interest, as well as the entire assembly, as a proxy for regions of low and high complexity. (PDF 4 kb) Additional file 4: Trimming length choice. Benchmarked universal single-copy ortholog (BUSCO) recovery from D*dn*-Anara assemblies trimmed at 1, 2, 3, 4, and 5 kb. The assembly trimmed at 2 kb was used for all downstream analyses. (PDF 5 kb) Additional file 5: Estimating true gap size in D*dn*-Anara. This table contains statistics on the size of gaps between D*dn*-Anara true contigs when the contigs are aligned to 3 different reference genomes. (PDF 4 kb) Additional file 6: Distribution of true gap size in D*dn*-Anara. This plot shows that the vast majority of gaps in D*dn*-Anara “scaffolds” are less than 1 kb. (PDF 241 kb) Additional file 7: Gaps in assemblies. This table contains statistics reported by the assembly evaluation tool GAEMR on gap sizes in AaraD1 and D*dn*-Anara. (PDF 4 kb) Additional file 8: Basic assembly statistics with increased coverage. Basic assembly statistics for DISCOVAR *de novo* assemblies made with high coverage (236× and 664×). Statistics for D*dn*-Anara, made with 121× coverage, are repeated from Tables 2 and 3 for reference. (PDF 6 kb) Additional file 9: Contiguity with increased coverage. Changes in numbers and distributions of AaraD1 contigs spanning multiple DISCOVAR *de novo*-produced contigs (oriented downward) and DISCOVAR *de novo*-produced contigs spanning multiple AaraD1 contigs (oriented upward). D*dn*-Anara, assembled from 121× coverage, is included for reference. Centromeres are marked with black circles. (PDF 617 kb) Additional file 10: Effects of increased coverage on contiguity in centromeric regions. A close-up of Additional file 9 in regions near the centromere of each chromosome. (PDF 237 kb) Additional file 11: Distribution of bubble lengths in D*dn*-Anara. This plot shows the frequency of bubble lengths from 1 to 100,000 bp, on a logarithmic scale. (PDF 92 kb) Additional file 12: Size distribution of separately assembled haplotypes. This table shows that the separately assembled haplotypes tend to be smaller than the rest of the assembly. (PDF 4 kb)

## Abbreviations

MR4: Malaria Research and Reference Reagent Resource Center
NIAID: National Institute of Allergy and Infectious Diseases
CTAB: cetyltrimethylammonium bromide
PCR: polymerase chain reaction
SNP: single nucleotide polymorphism
bp: base pair
kb: kilobase pairs
