# Supplementary material for: Evaluation of DISCOVAR de novo using a mosquito sample for cost-effective short-read genome assembly
Source: BMC Genomics. 2016 Mar 5;17:187. doi: 10.1186/s12864-016-2531-7 (PMC4779211; doi:10.1186/s12864-016-2531-7)
Supplement: Additional file 1: — Sequencing output. Approximate coverage for three versions of Ddn-Anara. (PDF 4 kb) [file 12864_2016_2531_MOESM1_ESM.pdf]

# reads	Mean Read Length	Coverage
114,798,132	250	120.9
229,596,264	250	236.2
669,875,594	250	664.1
