# Supplementary figures and images for: Evaluation of DISCOVAR de novo using a mosquito sample for cost-effective short-read genome assembly
Source: BMC Genomics. 2016 Mar 5;17:187. doi: 10.1186/s12864-016-2531-7 (PMC4779211; doi:10.1186/s12864-016-2531-7)

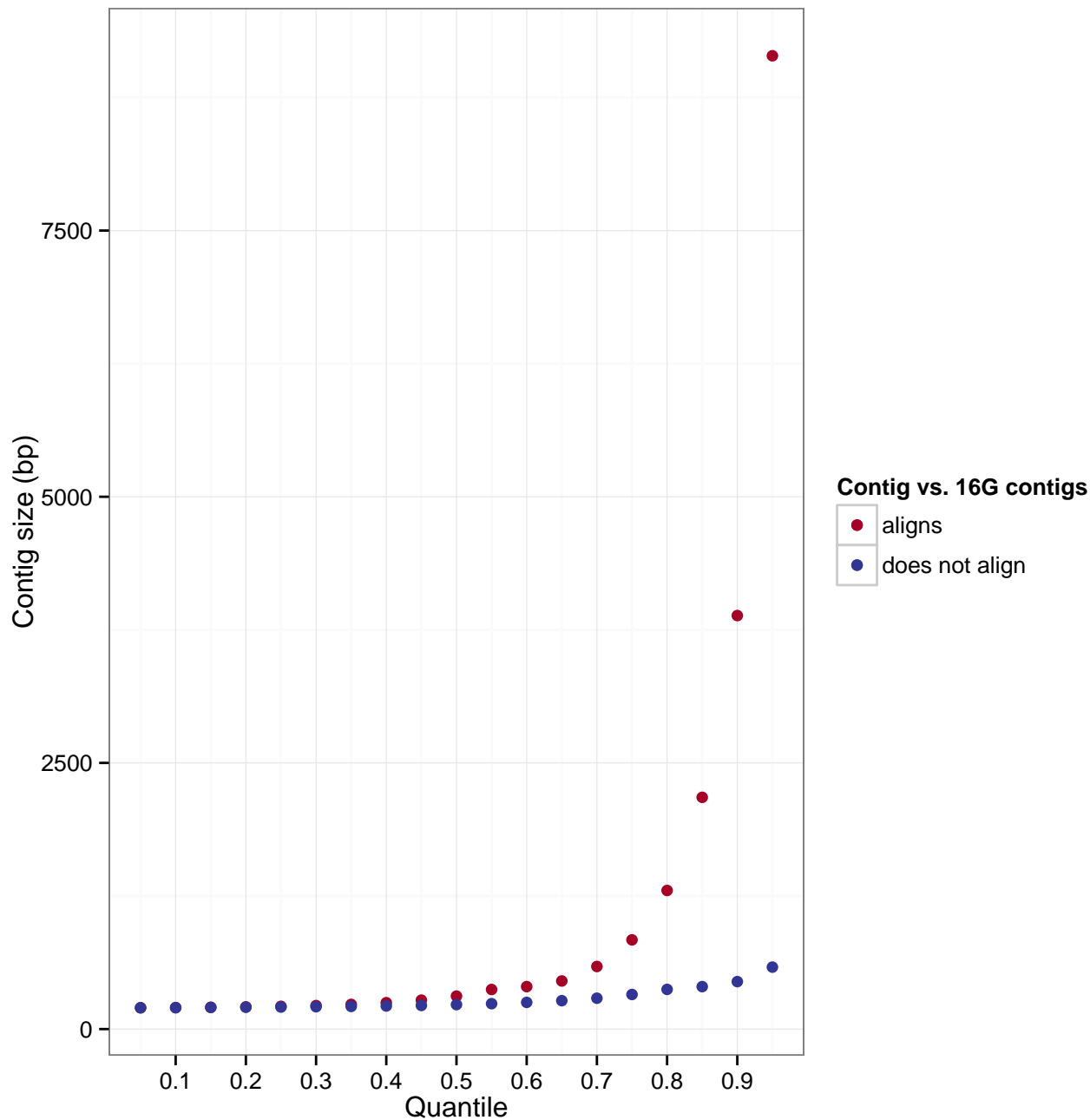

Supplement: Additional file 2: — Contig size vs. alignment status. This plot shows that the Ddn-Anara contigs that did not align to AaraD1 are some of the shortest in the assembly. (PDF 5 kb) [file 12864_2016_2531_MOESM2_ESM.pdf]

Distribution of gap sizes in *Ddn*-Anara contigs aligned to 3 references

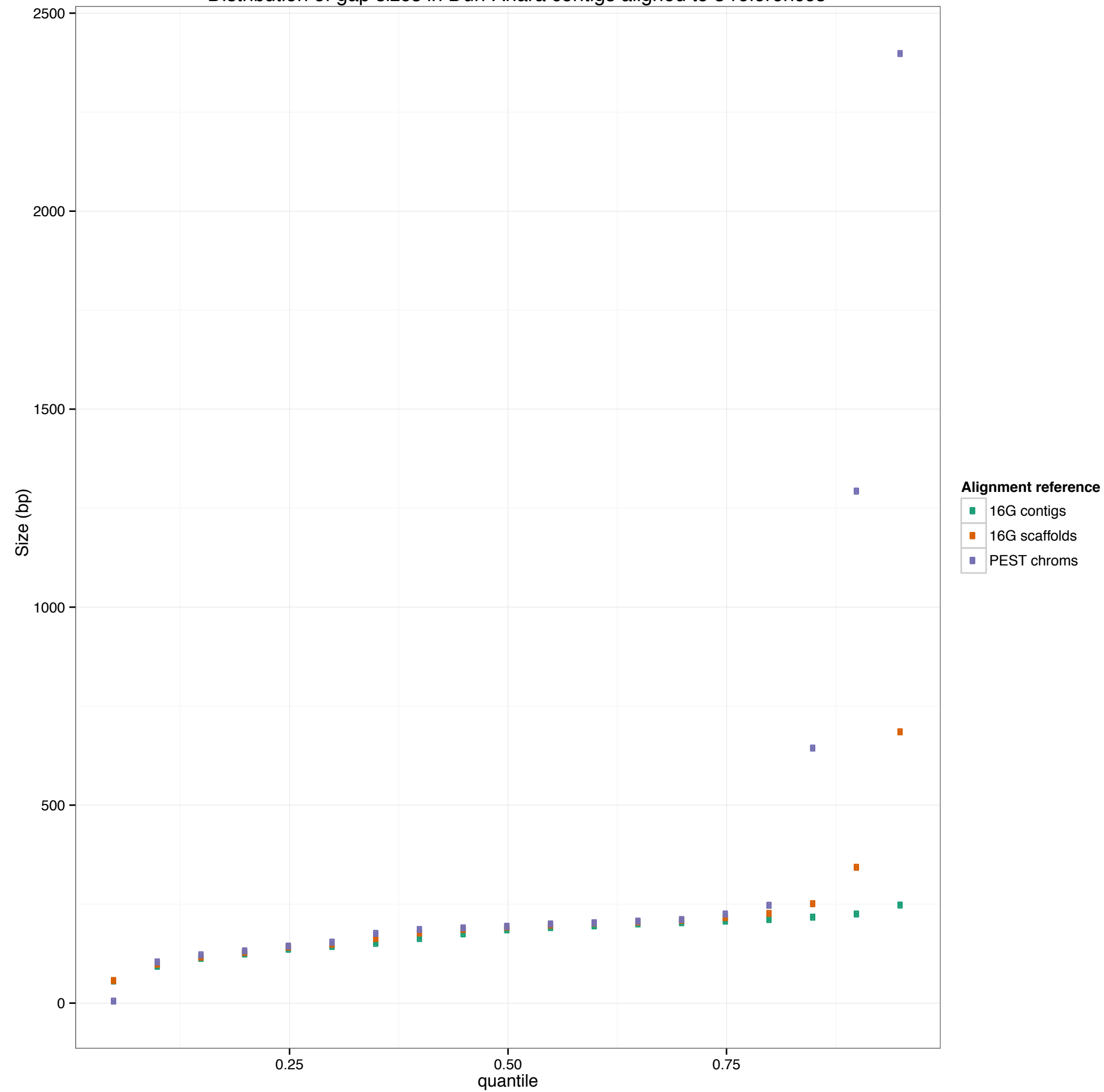

Supplement: Additional file 6: — Distribution of true gap size in Ddn-Anara. This plot shows that the vast majority of gaps in Ddn-Anara “scaffolds” are less than 1 kb. (PDF 241 kb) [file 12864_2016_2531_MOESM6_ESM.pdf]

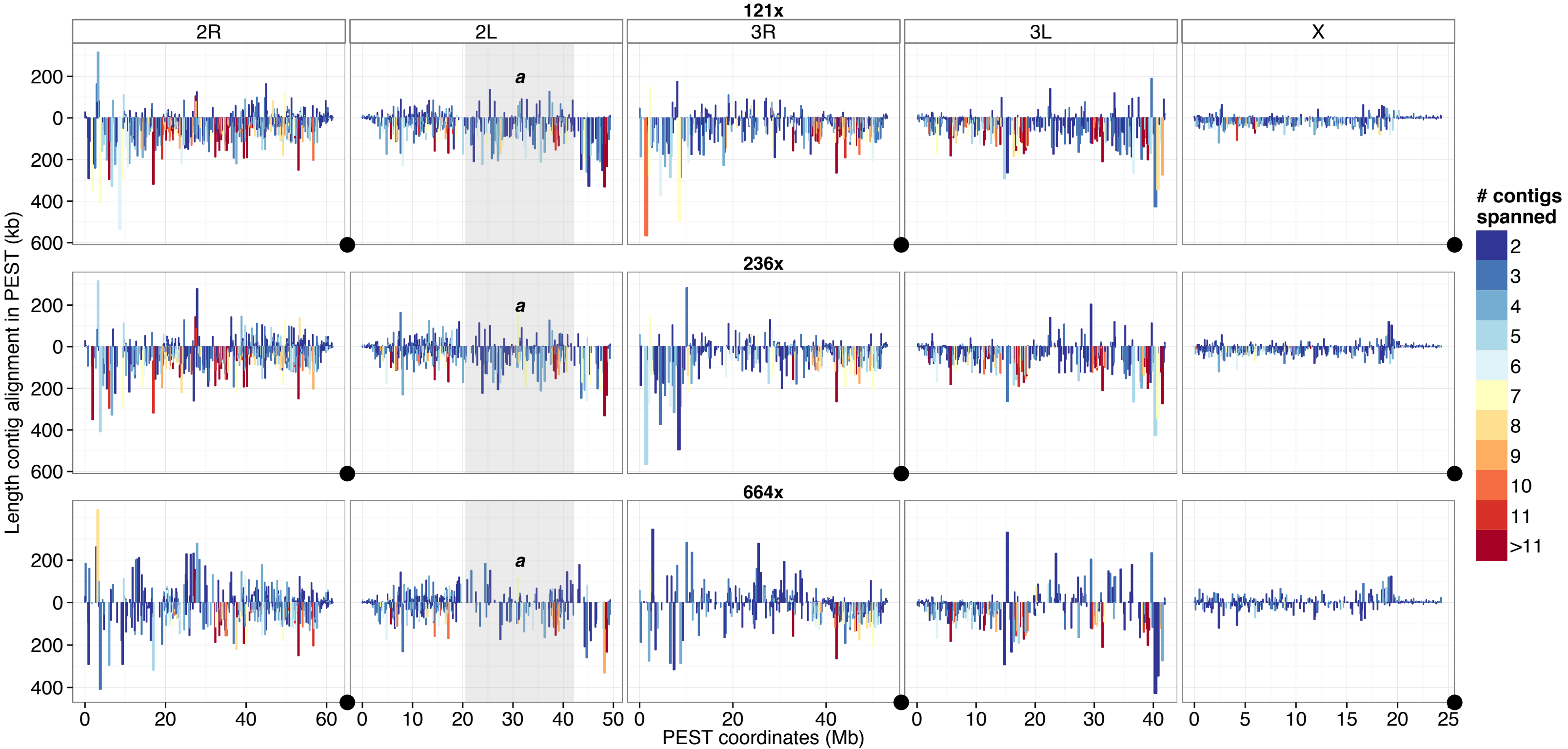

Supplement: Additional file 9: — Contiguity with increased coverage. Changes in numbers and distributions of AaraD1 contigs spanning multiple DISCOVAR de novo-produced contigs (oriented downward) and DISCOVAR de novo-produced contigs spanning multiple AaraD1 contigs (oriented upward). Ddn-Anara, assembled from 121× coverage, is included for reference. Centromeres are marked with black circles. (PDF 617 kb) [file 12864_2016_2531_MOESM9_ESM.pdf]

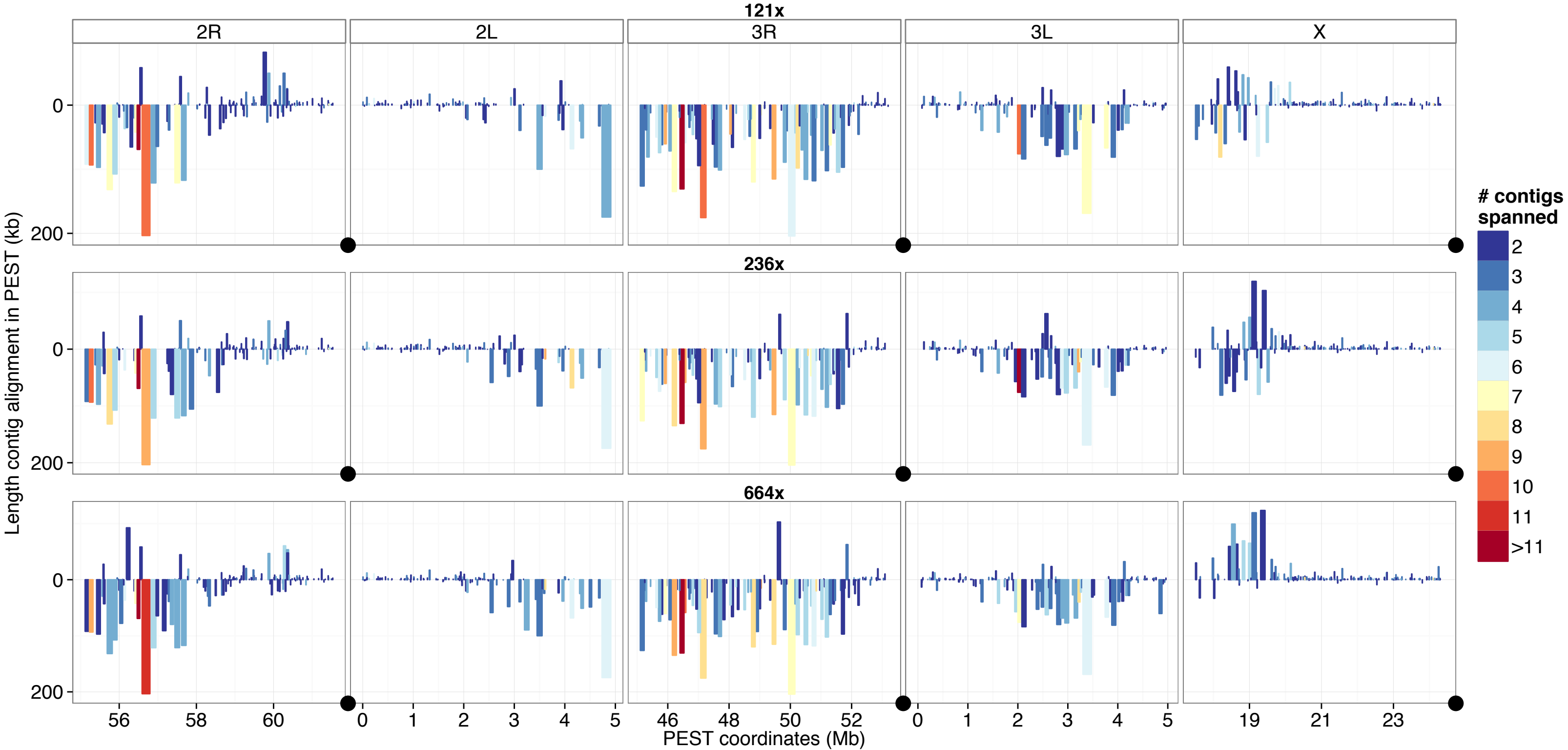

Supplement: Additional file 10: — Effects of increased coverage on contiguity in centromeric regions. A close-up of Additional file 9 in regions near the centromere of each chromosome. (PDF 237 kb) [file 12864_2016_2531_MOESM10_ESM.pdf]

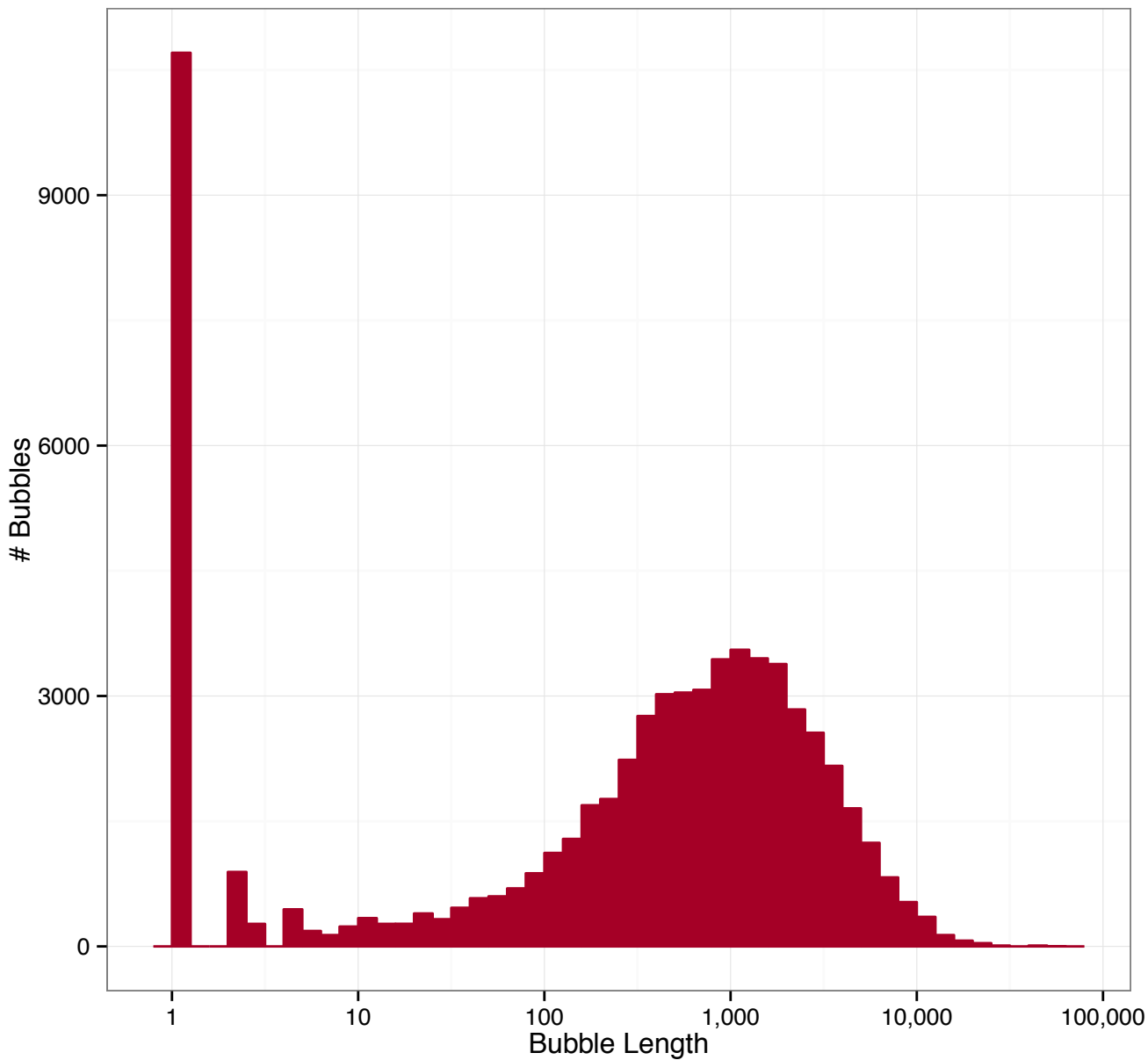

Supplement: Additional file 11: — Distribution of bubble lengths in Ddn-Anara. This plot shows the frequency of bubble lengths from 1 to 100,000 bp, on a logarithmic scale. (PDF 92 kb) [file 12864_2016_2531_MOESM11_ESM.pdf]
