# Supplementary material for: Evaluation of DISCOVAR de novo using a mosquito sample for cost-effective short-read genome assembly
Source: BMC Genomics. 2016 Mar 5;17:187. doi: 10.1186/s12864-016-2531-7 (PMC4779211; doi:10.1186/s12864-016-2531-7)
Supplement: Additional file 3: — Sequence complexity. This table shows the proportion of bases masked in regions of Ddn-Anara of particular interest, as well as the entire assembly, as a proxy for regions of low and high complexity. (PDF 4 kb) [file 12864_2016_2531_MOESM3_ESM.pdf]

Sequence	Total length	% GC content	% bases masked
AaraD1 inter−contig	1,036,206	41.86	16.57
Ddn−Anara	308,612,256	43.12	12.43
Ddn−Anara, contigs shorter than 2 kb	75,746,206	39.46	26.24
Ddn−Anara, trimmed at 2 kb	232,876,050	44.31	7.94
separately assembled haplotypes	3,833,901	42.82	8.48
