# Supplementary material for: Evaluation of DISCOVAR de novo using a mosquito sample for cost-effective short-read genome assembly
Source: BMC Genomics. 2016 Mar 5;17:187. doi: 10.1186/s12864-016-2531-7 (PMC4779211; doi:10.1186/s12864-016-2531-7)
Supplement: Additional file 4: — Trimming length choice. Benchmarked universal single-copy ortholog (BUSCO) recovery from Ddn-Anara assemblies trimmed at 1, 2, 3, 4, and 5 kb. The assembly trimmed at 2 kb was used for all downstream analyses. (PDF 5 kb) [file 12864_2016_2531_MOESM4_ESM.pdf]

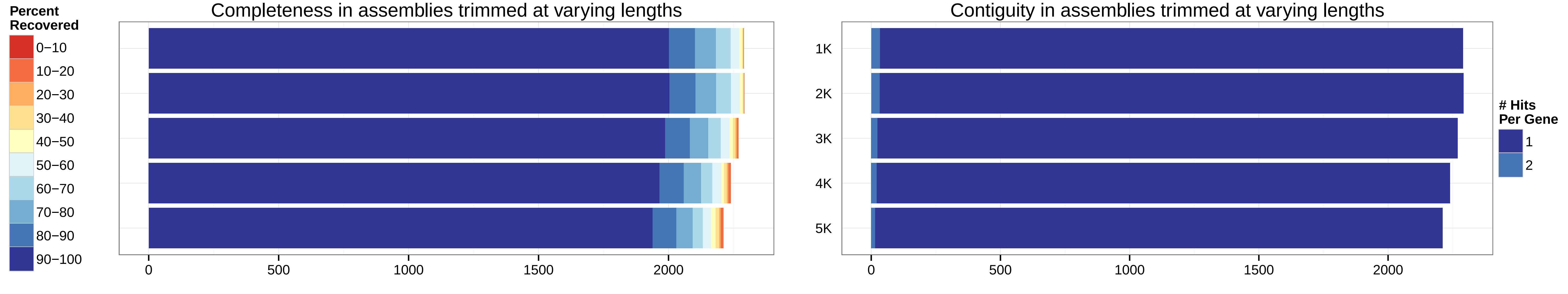
Percent Recovered
0−10
10−20
20−30
30−40
40−50
50−60
60−70
70−80
80−90
90−100
Completeness in assemblies trimmed at varying lengths
0
500
1000
1500
2000
Contiguity in assemblies trimmed at varying lengths
1K
2K
3K
4K
5K
0
500
1000
1500
2000
Hits Per Gene
1
2
