# Supplementary material for: Evaluation of DISCOVAR de novo using a mosquito sample for cost-effective short-read genome assembly
Source: BMC Genomics. 2016 Mar 5;17:187. doi: 10.1186/s12864-016-2531-7 (PMC4779211; doi:10.1186/s12864-016-2531-7)
Supplement: Additional file 5: — Estimating true gap size in Ddn-Anara. This table contains statistics on the size of gaps between Ddn-Anara true contigs when the contigs are aligned to 3 different reference genomes. (PDF 4 kb) [file 12864_2016_2531_MOESM5_ESM.pdf]

| Reference | Mean gap | Median gap | Minimum gap | Max gap | Gap length, 10th percentile | Gap length, 90th percentile |
|---|---|---|---|---|---|---|
| AaraD1 scaffolds | 256.20 | 192 | −89,471 | 20,021 | 98.2 | 343 |
| AaraD1 contigs | 56.86 | 185 | −89,471 | 6,443 | 93.0 | 225 |
| PEST chromosomes | 749.80 | 194 | −206 | 22,053 | 104.0 | 1,293 |
