# Supplementary material for: Evaluation of DISCOVAR de novo using a mosquito sample for cost-effective short-read genome assembly
Source: BMC Genomics. 2016 Mar 5;17:187. doi: 10.1186/s12864-016-2531-7 (PMC4779211; doi:10.1186/s12864-016-2531-7)
Supplement: Additional file 7: — Gaps in assemblies. This table contains statistics reported by the assembly evaluation tool GAEMR on gap sizes in AaraD1 and Ddn-Anara. (PDF 4 kb) [file 12864_2016_2531_MOESM7_ESM.pdf]

Assembly	# gaps	Max gap size	Mean gap size	Gap N50	Total gap length
AaraD1	8,965	36,105	3,918	9,460	35,124,750
Ddn−Anara, trimmed	1,656	100	100	100	165,600
