# Supplementary material for: Evaluation of DISCOVAR de novo using a mosquito sample for cost-effective short-read genome assembly
Source: BMC Genomics. 2016 Mar 5;17:187. doi: 10.1186/s12864-016-2531-7 (PMC4779211; doi:10.1186/s12864-016-2531-7)
Supplement: Additional file 8: — Basic assembly statistics with increased coverage. Basic assembly statistics for DISCOVAR de novo assemblies made with high coverage (236× and 664×). Statistics for Ddn-Anara, made with 121× coverage, are repeated from Tables 2 and 3 for reference. (PDF 6 kb) [file 12864_2016_2531_MOESM8_ESM.pdf]

Assembly	# Contigs	Contig N50	Contig N90	Mean contig length	Max contig length	Total contig length
Ddn−Anara	29,408	20,645	3,060	8,364	271,474	245,971,991
Ddn−Anara, trimmed	20,007	22,433	4,364	11,631	271,474	232,710,450
DISCOVAR de novo 236x	29,107	21,070	3,211	8,638	271,478	251,421,418
DISCOVAR de novo 236x, trimmed	19,982	22,879	4,551	11,939	271,478	238,569,130
DISCOVAR de novo 664x	26,121	29,719	3,620	9,945	406,289	259,767,112
DISCOVAR de novo 664x, trimmed	17,206	32,261	5,250	14,372	406,289	247,291,759

Assembly	# Scaffolds	Scaffold N50	Scaffold N90	Mean scaffold length	Max scaffold length	Total scaffold length	Read length	Insert size
Ddn−Anara	27,752	27,170	3,062	8,869	348,795	246,137,591	250	450
Ddn−Anara, trimmed	18,351	30,033	4,365	12,690	348,795	232,876,050	250	450
DISCOVAR de novo 236x	26,806	31,000	3,213	9,388	389,766	251,651,518	250	450
DISCOVAR de novo 236x, trimmed	17,681	34,315	4,552	13,506	389,766	238,799,230	250	450
DISCOVAR de novo 664x	24,142	46,579	3,623	10,768	684,404	259,965,012	250	450
DISCOVAR de novo 664x, trimmed	15,227	51,707	5,290	16,253	684,404	247,489,659	250	450
