# Supplementary material for: Evaluation of DISCOVAR de novo using a mosquito sample for cost-effective short-read genome assembly
Source: BMC Genomics. 2016 Mar 5;17:187. doi: 10.1186/s12864-016-2531-7 (PMC4779211; doi:10.1186/s12864-016-2531-7)
Supplement: Additional file 12: — Size distribution of separately assembled haplotypes. This table shows that the separately assembled haplotypes tend to be smaller than the rest of the assembly. (PDF 4 kb) [file 12864_2016_2531_MOESM12_ESM.pdf]

	0%	10%	20%	30%	40%	50%	60%	70%	80%	90%	100%
all contigs	2,000	2,346	2,810	3,370	4,115	5,124	6,731	9,664	15,333	30,187	348,795
separately assembled haplotypes	2,007	2,215	2,426	2,675	2,961	3,215	3,668	4,168	4,920	6,286	16,201
